# The Truncated Burr X-G Family of Distributions: Properties and Applications to Actuarial and Financial Data

**DOI:** 10.3390/e23081088

**Published:** 2021-08-21

**Authors:** Rashad A. R. Bantan, Christophe Chesneau, Farrukh Jamal, Ibrahim Elbatal, Mohammed Elgarhy

**Affiliations:** 1 Department of Marine Geology, Faculty of Marine Science, King AbdulAziz University, Jeddah 21551, Saudi Arabia; rbantan@kau.edu.sa; 2 Department of Mathematics, Université de Caen, LMNO, Campus II, Science 3, 14032 Caen, France; 3 Department of Statistics, The Islamia University of Bahawalpur, Bahawalpur 63100, Pakistan; Farrukh.jamal@iub.edu.pk; 4 Department of Mathematics and Statistics, College of Science, Imam Mohammad Ibn Saud Islamic University (IMSIU), Riyadh 11432, Saudi Arabia; iielbatal@imamu.edu.sa; 5 The Higher Institute of Commercial Sciences, Al mahalla Al kubra, Algarbia 31951, Egypt; m_elgarhy85@sva.edu.eg

**Keywords:** truncated distributions, Burr X distribution, general family of distributions, moments, risk measures, maximum likelihood technique, data analysis

## Abstract

In this article, the “truncated-composed” scheme was applied to the Burr X distribution to motivate a new family of univariate continuous-type distributions, called the truncated Burr X generated family. It is mathematically simple and provides more modeling freedom for any parental distribution. Additional functionality is conferred on the probability density and hazard rate functions, improving their peak, asymmetry, tail, and flatness levels. These characteristics are represented analytically and graphically with three special distributions of the family derived from the exponential, Rayleigh, and Lindley distributions. Subsequently, we conducted asymptotic, first-order stochastic dominance, series expansion, Tsallis entropy, and moment studies. Useful risk measures were also investigated. The remainder of the study was devoted to the statistical use of the associated models. In particular, we developed an adapted maximum likelihood methodology aiming to efficiently estimate the model parameters. The special distribution extending the exponential distribution was applied as a statistical model to fit two sets of actuarial and financial data. It performed better than a wide variety of selected competing non-nested models. Numerical applications for risk measures are also given.

## 1. Introduction

In most branches of applied sciences, the practitioner must process data to understand all aspects of this object of study. The use of efficient statistical models is often a minimum condition to achieve this goal. These models are based on mathematical representations, which can be of various kinds, depending on the context and objective. One type of useful model is based on parametric distributions with desirable properties. Logically, the more flexible the distribution in terms of functionality, the more efficient the associated model is for data analysis. To be usable, the distributions must also be of moderate complexity, which depends mainly on their number of parameters and the nature of the involved functions, even if the development of computer science has relaxed this constraint a little.

Since the turn of the millennium, a modern approach has sought to create general families of continuous distributions that generalize or extend any referenced distribution, referred to as the parental, baseline, or reference distribution, using specific mathematical schemes. These schemes consist of transforming the parental cumulative distribution function (cdf) via parametric functions. New shape or scale parameters are often involved in this analytical operation. An overview can be found in [1,2,3]. A list of recent families of distributions is the following: truncated Fréchet generated (TF-G) family by [4], truncated Weibull generated (TW-G) family by [5], truncated inverted Kumaraswamy generated (TIK-G) family by [6], type II truncated Fréchet generated (TIITF-G) family by [7], transmuted Muth generated (TM-G) family by [8], Box–Cox gamma generated (BCG-G) family by [9], Topp–Leone odd Fréchet generated (TLOF-G) family by [10], exponentiated power generalized Weibull power series generated (EPGWPS-G) family by [11], xgamma generated (XG-G) family by [12], truncated Cauchy power generated (TCP-G) family by [13], exponentiated truncated inverse Weibull generated (ETIW-G) family by [14], transmuted odd Fréchet generated (TOF-G) family by [15], Marshall–Olkin exponentiated generalized generated (MOEG-G) family by [16], exponentiated M generated (EM-G) family by [17], type II power Topp–Leone generated (TIITL-G) family by [18], modified T-X generated (MTX-G) family by [19], modified odd Weibull generated (MOW-G) family by [20], truncated Burr generated (TB-G) family by [21], type II general inverse exponential generated (TIIGIE-G) family by [22], odd generalized gamma generated (OGG-G) family by [23], and truncated generalized Fréchet generated (TGF-G) family by [24].

Some of these families are constructed from the “truncation-composition” scheme, such as the TF-G, TIK-G, TIITF-G, TCP-G, ETIW-G, TB-G, and TGF-G families mentioned above. This scheme is based on the following process: first, truncate the cdf of a flexible survival distribution over the unit interval, then compose it with a cdf of an arbitrary parental distribution. Its main interest is to create families capable of producing simple and original distribution functions with new modeling potential. Of course, the characteristics and practical aspects of the family mainly depend on the choices of the cdfs involved. This “truncation-composition” scheme will be at the center of the proposed methodology.

On another plan, the Burr X (BX) distribution was invented and developed by [25] as a special survival function. From the mathematical point of view, its two-parameter version is listed by the following cdf:

$$\begin{array}{c} F_{BX}\left(x;\alpha ,\theta \right)={\left[1-e^{-{\left(\alpha x\right)}^{2}}\right]}^{\theta },\;x>0,\alpha >0,\theta >0, \end{array}$$

and $F_{BX}\left(x;\alpha ,\theta \right)=0$ for x≤0, where α and θ are scale and shape parameters, respectively. Furthermore, the associated pdf is given by:

$$\begin{array}{c} f_{BX}\left(x;\alpha ,\theta \right)=2\alpha^{2}\theta xe^{-{\left(\alpha x\right)}^{2}}{\left[1-e^{-{\left(\alpha x\right)}^{2}}\right]}^{\theta -1},\;x>0, \end{array}$$

and $f_{BX}\left(x;\alpha ,\theta \right)=0$ for x≤0. Owing to a wise compromise between simplicity and flexibility, the BX distribution has received much attention. Important references in this regard include [26,27,28,29,30,31,32,33,34]. The BX distribution has been proven to be a proper alternative to the weighted exponential, exponentiated exponential, gamma, and Weibull distributions in some data analysis problems. It finds many applications in reliability, agriculture, engineering, and biology. The BX distribution is also the main ingredient of the BX generated (BX-G) family studied in depth by [35]. It is constructed from the “odd-composition” scheme; the BX-G family is registered by the following cdf:

$$\begin{array}{cc} F_{BX-G}\left(x;\alpha ,\theta \right) & =F_{BX}\left(\frac{G(x;\omega )}{\bar{G}\left(x;\omega \right)};\alpha ,\theta \right)\\ \; & ={\left[1-e^{-{\left(\alpha G\left(x;\omega \right)/\bar{G}\left(x;\omega \right)\right)}^{2}}\right]}^{\theta },\;x\in R, \end{array}$$

where G(x;ω) is a cdf of a parental distribution with a parameter vector ω and $\bar{G}\left(x;\omega \right)=1-G\left(x;\omega \right)$. It has been proven that the functionalities of the BX-G family have the power to enhance the modeling capabilities of the parental distribution (see [35,36]).

In this study, we employed the BX distribution for further modeling and application perspectives. In light of the paragraphs above, we propose to apply the “truncation-composition” scheme to the BX distribution to develop a new family. Its detailed construction and main motivations are described below. First, we considered the truncated version of $F_{BX}\left(x;\alpha ,\theta \right)$ over the unit interval given as:

(1) $$\begin{array}{cc} F_{TBX}\left(x;\alpha ,\theta \right) & =\frac{F_{BX}\left(x;\alpha ,\theta \right)}{F_{BX}\left(1;\alpha ,\theta \right)}\\ \; & =\frac{1}{{\left(1-e^{-\alpha^{2}}\right)}^{\theta }}{\left[1-e^{-{\left(\alpha x\right)}^{2}}\right]}^{\theta },\;x\in \left(0,1\right), \end{array}$$

$F_{TBX}\left(x;\alpha ,\theta \right)=0$ for x<0 and $F_{TBX}\left(x;\alpha ,\theta \right)=1$ for x>1. Then, upon the composition of Equation (1) with the cdf of a parental distribution, we define the truncated BX generated (TBX-G) family by the following cdf:

(2) $$\begin{array}{cc} F_{TBX-G}\left(x;\alpha ,\theta ,\omega \right) & =F_{TBX}\left(G\left(x;\omega \right);\alpha ,\theta \right)\\ \; & =\frac{1}{{\left(1-e^{-\alpha^{2}}\right)}^{\theta }}{\left[1-e^{-{\left(\alpha G(x;\omega )\right)}^{2}}\right]}^{\theta },\;x\in R. \end{array}$$

At first glance, we can notice the manageability of $F_{TBX-G}\left(x;\alpha ,\theta ,\omega \right)$. From a purely analytical point of view, it seems easier to use than the cdf of the BX-G family, which deals with the possibly complex ratio $G\left(x;\omega \right)/\bar{G}\left(x;\omega \right)$. Secondly, we underline its novelty: to our knowledge, it has not been studied in any form in the existing literature. On the other hand, the TBX-G family has some interesting connections with well-reputed families. When α tends to zero, $F_{TBX-G}\left(x;\alpha ,\theta ,\omega \right)$ tends to $G{\left(x;\omega \right)}^{2\theta }$, which defines the famous exponentiated generated (E-G) family with parameter 2θ. In addition, the TBX-G and TW-G families coincide under the following configuration: θ=1 for the TBX-G family, and for the TW-G family, the scale parameter is equal to $\alpha^{2}$ and the shape parameter to two. The success of the TW-G family in applications is a motivation for more work on the TBX-G family.

In this study, we develop the TBX-G family, highlighting its richness and potential for applicability. In the first part, we introduce the main functions of the TBX-G family and, in particular, the pdf, hazard rate function (hrf) and quantile function (qf). Then, three special members of the family based on the exponential, Rayleigh, and Lindley distributions are discussed. A battery of general properties of the TBX-G family is examined, including the asymptotic action of the pdf, hrf, and qf, stochastic dominance results on the cdf, series expansion of the exponentiated pdf, Tsallis entropy measure, and moment-type measures. A paragraph concerns useful risk measures, with a focus on the value at risk and expected shortfall. Then, statistical uses of the special model extending the exponential distribution are developed through the maximum likelihood (ML) technique. It is applied to fit two sets of actuarial and financial data. Using standard benchmarks, we reveal that it performs better than eight selected competing models. Then, the estimation of risk measures for the considered datasets is discussed, with a fairly satisfactory result for the proposed methodology.

The following sections compose the article. Section 2 exhibits the pdf and hrf of the TBX-G family, with illustrations of the three mentioned special distributions. Section 3 is dedicated to the mathematical properties. Section 4 develops the corresponding statistical and inferential approaches. Section 5 contains two applications for data. Section 6 is conclusive; some endnotes and perspectives are formulated.

## 2. Presentation of the TBX-G Family

The TBX-G family is first and foremost characterized by the cdf given in Equation (2). Other functions of interest can be expressed and discussed to capture the modeling capacity of the family. This section presents these functions, with concrete examples.

### 2.1. Distributional Functions

Here, the general expressions of the pdf, hrf, and qf are given.

#### 2.1.1. Definition of the pdf

The pdf of the TBX-G family is derived from Equation (2) by differentiation; after elementary operations, we obtain:

(3) $$\begin{array}{cc} f_{TBX-G}\left(x;\alpha ,\theta ,\omega \right) & =\frac{2\theta \alpha^{2}}{{\left(1-e^{-\alpha^{2}}\right)}^{\theta }}g\left(x;\omega \right)G\left(x;\omega \right)e^{-{\left(\alpha G(x;\omega )\right)}^{2}}{\left[1-e^{-{\left(\alpha G(x;\omega )\right)}^{2}}\right]}^{\theta -1},\\ \; & \;x\in R, \end{array}$$

where g(x;ω) is the pdf derived from G(x;ω). Classically, for a random variable *X* with pdf $f_{TBX-G}\left(x;\alpha ,\theta ,\omega \right)$ and any function r(x), we have:

(4) $$\begin{array}{c} E\left[r\left(X\right)\right]=\int_{R}r\left(x\right)f_{TBX-G}\left(x;\alpha ,\theta ,\omega \right)dx, \end{array}$$

where *E* stands for expectation, provided the integral exists. Specific choices for r(x) define useful probabilistic measures and functions. Some of them will be discussed later.

#### 2.1.2. Definition of the hrf

Based on the functions in Equations (2) and (3), the survival analysis of the TBX-G family can be performed through the hrf defined by:

(5) $$\begin{array}{cc} \; & h_{TBX-G}\left(x;\alpha ,\theta ,\omega \right)=\frac{f_{TBX-G}\left(x;\alpha ,\theta ,\omega \right)}{1-F_{TBX-G}\left(x;\alpha ,\theta ,\omega \right)}\\ \; & =\frac{2\theta \alpha^{2}g\left(x;\omega \right)}{{\left(1-e^{-\alpha^{2}}\right)}^{\theta }-{\left[1-e^{-{\left(\alpha G(x;\omega )\right)}^{2}}\right]}^{\theta }}G\left(x;\omega \right)e^{-{\left(\alpha G(x;\omega )\right)}^{2}}{\left[1-e^{-{\left(\alpha G(x;\omega )\right)}^{2}}\right]}^{\theta -1},\;x\in R. \end{array}$$

This function takes its full sense where the reference distribution is a survival distribution. In this case, it finds numerous applications in various fields, such as in demography and actuarial science, where the hrf represents the force of mortality. From the analytical point of view, the curvature of the hrf can be of various forms: it can be monotonic, not monotonic, or discontinuous. The diverse meanings of this diversity of forms can be found in [37].

#### 2.1.3. Definition of the qf

The qf of the TBX-G family is derived to Equation (2) by the standard inversion technique; after elementary developments, we obtain:

(6) $$\begin{array}{cc} Q_{TBX-G}\left(u;\alpha ,\theta ,\omega \right) & =F_{TBX-G}^{-1}\left(u;\alpha ,\theta ,\omega \right)\\ \; & =Q_{G}\left(\frac{1}{\alpha }{\left\{-\log\left[1-u^{1/\theta }\left(1-e^{-\alpha^{2}}\right)\right]\right\}}^{1/2},\omega \right),\;u\in \left(0,1\right), \end{array}$$

where $Q_{G}\left(u;\omega \right)=G^{-1}\left(u;\omega \right)$. The qf is very useful in probability and statistics, allowing the definitions of various measures and functions. The importance of quantile measures/functions was highlighted in [38]. In order to understand the effects of the shape parameters on the skewness and kurtosis of the TBX-G family, one can use the Bowley skewness measure defined by:

(7) $$\begin{array}{c} SK=\frac{Q_{TBX-G}\left(3/4;\alpha ,\theta ,\omega \right)+Q_{TBX-G}\left(1/4;\alpha ,\theta ,\omega \right)-2Q_{TBX-G}\left(1/2;\alpha ,\theta ,\omega \right)}{Q_{TBX-G}\left(3/4;\alpha ,\theta ,\omega \right)-Q_{TBX-G}\left(1/4;\alpha ,\theta ,\omega \right)} \end{array}$$

and the Moors kurtosis measure defined by:

(8) $$\begin{array}{c} KU=\frac{Q_{TBX-G}\left(7/8;\alpha ,\theta ,\omega \right)-Q_{TBX-G}\left(5/8;\alpha ,\theta ,\omega \right)+Q_{TBX-G}\left(3/8;\alpha ,\theta ,\omega \right)-Q_{TBX-G}\left(1/8;\alpha ,\theta ,\omega \right)}{Q_{TBX-G}\left(6/8;\alpha ,\theta ,\omega \right)-Q_{TBX-G}\left(2/8;\alpha ,\theta ,\omega \right)}. \end{array}$$

The main advantages of SK and KU are their mathematical existence under all circumstances and simplicity. In the case of unimodal distributions, a positive KU indicates heavy tails and peakedness, whereas a negative KU indicates light tails and flatness.

The presented pdf, hrf, and qf are fundamental for further statistical processing of the TBX-G family, and their relative simplicity motivates more in this regard.

### 2.2. Special Survival Distributions

Here, we illustrate the modeling richness of the TBX-G family by introducing three of its survival distributions. The following parental distributions are considered: exponential, Rayleigh, and Lindley distributions.

#### 2.2.1. TBX Exponential Distribution

**Figure 1:**
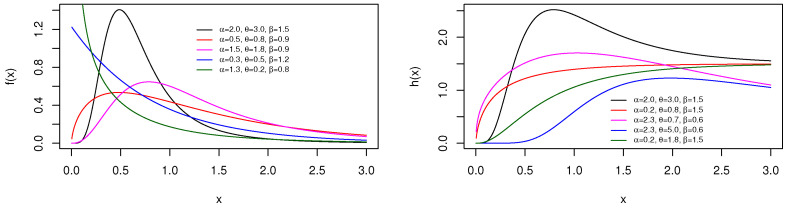
Plots of the pdf (**left**) and hrf (**right**) of the TBXE distribution for specific parameter values.

The TBX exponential (TBXE) distribution is a three-parameter survival member of the TBX-G family. It is defined with the exponential distribution as a parent, that is ω=(β), $G_{E}\left(x;\beta \right)=1-e^{-\beta x}$ for x>0 and $G_{E}\left(x;\beta \right)=0$ for x≤0 and $g_{E}\left(x;\beta \right)=\beta e^{-\beta x}$ for x>0 and $g_{E}\left(x;\beta \right)=0$ for x≤0. Hence, based on Equations (2), (3), and (5), the cdf, pdf, and hrf of the TBXE distribution are:

$$F_{TBXE}\left(x;\alpha ,\theta ,\beta \right)=\frac{1}{{\left(1-e^{-\alpha^{2}}\right)}^{\theta }}{\left\{1-e^{-{\left[\alpha (1-e^{-\beta x})\right]}^{2}}\right\}}^{\theta },\;x>0,$$

and $F_{TBXE}\left(x;\alpha ,\theta ,\beta \right)=0$ for x≤0,

$$\begin{array}{cc} f_{TBXE}\left(x;\alpha ,\theta ,\beta \right) & =\frac{2\theta \alpha^{2}\beta }{{\left(1-e^{-\alpha^{2}}\right)}^{\theta }}e^{-\beta x}\left(1-e^{-\beta x}\right)e^{-{\left(\alpha (1-e^{-\beta x})\right)}^{2}}{\left\{1-e^{-{\left[\alpha (1-e^{-\beta x})\right]}^{2}}\right\}}^{\theta -1},\\ \; & x>0, \end{array}$$

and $f_{TBXE}\left(x;\alpha ,\theta ,\beta \right)=0$ for x≤0 and

$$\begin{array}{cc} h_{TBXE}\left(x;\alpha ,\theta ,\beta \right) & =\frac{2\theta \alpha^{2}\beta }{{\left(1-e^{-\alpha^{2}}\right)}^{\theta }-{\left\{1-e^{-{\left[\alpha (1-e^{-\beta x})\right]}^{2}}\right\}}^{\theta }}e^{-\beta x}\left(1-e^{-\beta x}\right)e^{-{\left[\alpha (1-e^{-\beta x})\right]}^{2}}\times \\ \; & {\left\{1-e^{-{\left[\alpha (1-e^{-\beta x})\right]}^{2}}\right\}}^{\theta -1},\;x>0, \end{array}$$

and $h_{TBXE}\left(x;\alpha ,\theta ,\beta \right)=0$ for x≤0, respectively. A visual approach of the pdf and hrf is offered in Figure 1, showing the diversity of shapes possessed by these functions at chosen values of the parameters.

Figure 1 reveals that the pdf of the TBXE distribution can be decreasing or unimodal and right-skewed with various roundness, tail, and asymmetric properties. For its part, the hrf presents a wide variety of increasing and unimodal shapes. It is clear that the TBXE distribution is more flexible than the exponential distribution; the “truncated-composed BX scheme” has enriched the catalog of shapes of the basic exponential distribution.

A quantile analysis of the TBXE distribution is now afforded. Based on Equation (6), the qf of the TBXE distribution is:

(9) $$\begin{array}{cc} Q_{TBXE}\left(u;\alpha ,\theta ,\beta \right) & =-\frac{1}{\beta }\log\left(1-\frac{1}{\alpha }{\left\{-\log\left[1-u^{1/\theta }\left(1-e^{-\alpha^{2}}\right)\right]\right\}}^{1/2}\right),\;u\in \left(0,1\right). \end{array}$$

**Figure 2:**
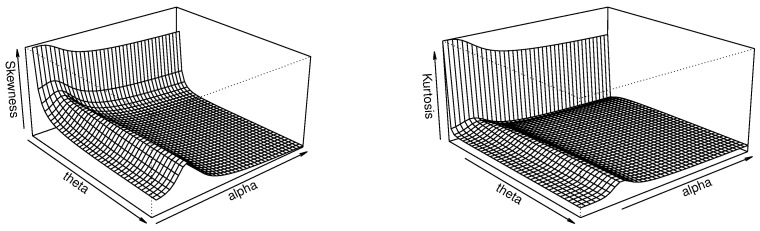
Three-dimensional plots of SK (**left**) and KU (**right**) for the TBXE distribution for β=1.

Thanks to this closed-form expression, we can express several quantile measures, such as SK and KU, as described in Equations (7) and (8), respectively. Three-dimensional plots of SK and KU are proposed in Figure 2 for a fixed value of β and varying values for α and θ.

Figure 2 indicates that, for the fixed value β=1, both the skewness and kurtosis of the TBXE distribution are decreasing in θ and unimodal with respect to α. This reflects a certain flexibility in these aspects, which is beneficial for modeling objectives.

#### 2.2.2. TBX Rayleigh Distribution

**Figure 3:**
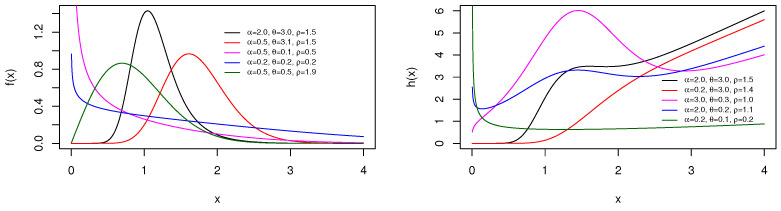
Plots of the pdf (**left**) and hrf (**right**) for the TBXR distribution for some parameter values.

Another member of interest in the TBX-G family is the TBX Rayleigh (TBXR) distribution, which considers the Rayleigh distribution as a parent. Therefore, we set ω=(ρ), $G_{R}\left(x;\rho \right)=1-e^{-\rho x^{2}/2}$ for x>0 and $G_{R}\left(x;\rho \right)=0$ for x≤0 and $g_{R}\left(x;\rho \right)=\rho xe^{-\rho x^{2}/2}$ for x>0 and $g_{R}\left(x;\rho \right)=0$ for x≤0. By virtue of Equations (2), (3), and (5), the cdf, pdf, and hrf of the TBXR distribution are:

$$F_{TBXR}\left(x;\alpha ,\theta ,\rho \right)=\frac{1}{{\left(1-e^{-\alpha^{2}}\right)}^{\theta }}{\left\{1-e^{-{\left[\alpha \left(1-e^{-\rho x^{2}/2}\right)\right]}^{2}}\right\}}^{\theta },\;x>0,$$

and $F_{TBXR}\left(x;\alpha ,\theta ,\rho \right)=0$ for x≤0,

$$\begin{array}{cc} f_{TBXR}\left(x;\alpha ,\theta ,\rho \right) & =\frac{2\theta \alpha^{2}\rho }{{\left(1-e^{-\alpha^{2}}\right)}^{\theta }}xe^{-\rho x^{2}/2}\left(1-e^{-\rho x^{2}/2}\right)e^{-{\left[\alpha \left(1-e^{-\rho x^{2}/2}\right)\right]}^{2}}{\left\{1-e^{-{\left[\alpha \left(1-e^{-\rho x^{2}/2}\right)\right]}^{2}}\right\}}^{\theta -1},\\ \; & \;x>0, \end{array}$$

and $f_{TBXR}\left(x;\alpha ,\theta ,\rho \right)=0$ for x≤0 and

$$\begin{array}{cc} h_{TBXR}\left(x;\alpha ,\theta ,\rho \right) & =\frac{2\theta \alpha^{2}\rho }{{\left(1-e^{-\alpha^{2}}\right)}^{\theta }-{\left\{1-e^{-{\left[\alpha \left(1-e^{-\rho x^{2}/2}\right)\right]}^{2}}\right\}}^{\theta }}xe^{-\rho x^{2}/2}\left(1-e^{-\rho x^{2}/2}\right)e^{-{\left[\alpha \left(1-e^{-\rho x^{2}/2}\right)\right]}^{2}}\times \\ \; & {\left\{1-e^{-{\left[\alpha \left(1-e^{-\rho x^{2}/2}\right)\right]}^{2}}\right\}}^{\theta -1},\;x>0, \end{array}$$

and $h_{TBXR}\left(x;\alpha ,\theta ,\rho \right)=0$ for x≤0, respectively. Figure 3 shows a sample of shapes reached by the pdf and hrf by considering some parameter values.

From Figure 3, we see that the pdf of the TBXR distribution can be decreasing or unimodal with an “almost symmetrical bell shape” and right-skewed. Furthermore, the hrf curves can be decreasing, increasing, and N shapes. This panel of shapes is not observed with the Rayleigh distribution.

Based on Equation (6), the qf of the TBXR distribution is:

(10) $$\begin{array}{cc} Q_{TBXR}\left(u;\alpha ,\theta ,\rho \right) & ={\left\{-\frac{2}{\rho }\log\left(1-\frac{1}{\alpha }{\left\{-\log\left[1-u^{1/\theta }\left(1-e^{-\alpha^{2}}\right)\right]\right\}}^{1/2}\right)\right\}}^{1/2},\;u\in \left(0,1\right). \end{array}$$

The skewness and kurtosis analyses of the TBXR distribution are performed via the quantile measures SK and KU as defined by Equations (7) and (8), respectively, in Figure 2.

**Figure 4:**
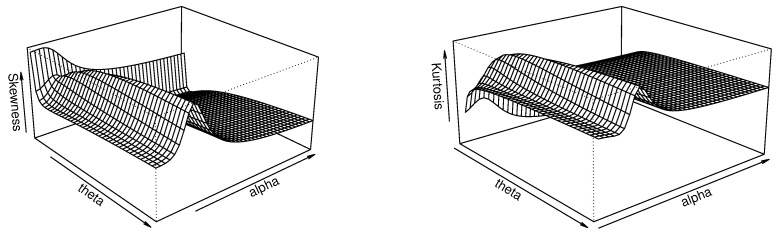
Three-dimensional plots of SK (**left**) and KU (**right**) of the TBXR distribution for ρ=1.

Figure 4 indicates that, for the fixed value ρ=1, the skewness of the TBXR distribution is unimodal in θ and α, while the kurtosis is increasing in θ and unimodal in α. This behavior, quite complex, remains an advantage of the TBXR model to adapt to various kinds of data.

#### 2.2.3. TBX Lindley Distribution

**Figure 5:**
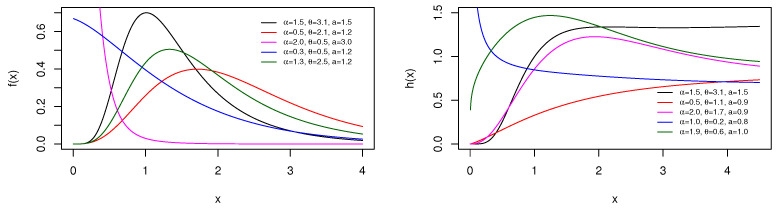
Plots of the pdf (**left**) and hrf (**right**) of the TBXL distribution for chosen parameter values.

We now present the TBX Lindley (TBXL) distribution, corresponding to the member of the TBX-G family defined via the Lindley distribution. Hence, we set ω=(a), $G_{L}\left(x;a\right)=1-\left[1+ax/\left(1+a\right)\right]e^{-ax}$ for x>0 and $G_{L}\left(x;a\right)=0$ for x≤0 and $g_{L}\left(x;a\right)=a^{2}\left(1+x\right)e^{-ax}/\left(1+a\right)$ for x>0 and $g_{L}\left(x;a\right)=0$ for x≤0. Hence, by virtue of Equations (2), (3), and (5), the cdf, pdf, and hrf of the TBXL distribution are:

$$F_{TBXL}\left(x;\alpha ,\theta ,a\;\right)=\frac{1}{{\left(1-e^{-\alpha^{2}}\right)}^{\theta }}{\left\{1-e^{-{\left[\alpha \left(1-\left[1+ax/\left(1+a\right)\right]e^{-ax}\right)\right]}^{2}}\right\}}^{\theta },\;x>0,$$

and $F_{TBXL}\left(x;\alpha ,\theta ,a\;\right)=0$ for x≤0,

$$\begin{array}{cc} f_{TBXL}\left(x;\alpha ,\theta ,a\;\right) & =\frac{2\theta \alpha^{2}a^{2}}{\left(1+a\right){\left(1-e^{-\alpha^{2}}\right)}^{\theta }}\left(1+x\right)e^{-ax}\left(1-\left[1+\frac{ax}{1+a}\right]e^{-ax}\right)e^{-{\left[\alpha \left(1-\left[1+ax/\left(1+a\right)\right]e^{-ax}\right)\right]}^{2}}\times \\ \; & {\left\{1-e^{-{\left[\alpha \left(1-\left[1+ax/\left(1+a\right)\right]e^{-ax}\right)\right]}^{2}}\right\}}^{\theta -1},\;x>0, \end{array}$$

and $f_{TBXL}\left(x;\alpha ,\theta ,a\right)=0$ for x≤0 and

$$\begin{array}{cc} h_{TBXL}\left(x;\alpha ,\theta ,a\;\right) & =\frac{2\theta \alpha^{2}a^{2}}{\left(1+a\right)\left[{\left(1-e^{-\alpha^{2}}\right)}^{\theta }-{\left\{1-e^{-{\left[\alpha \left(1-\left[1+ax/\left(1+a\right)\right]e^{-ax}\right)\right]}^{2}}\right\}}^{\theta }\right]}\left(1+x\right)e^{-ax}\times \\ & \left(1-\left[1+\frac{ax}{1+a}\right]e^{-ax}\right)e^{-{\left[\alpha \left(1-\left[1+ax/\left(1+a\right)\right]e^{-ax}\right)\right]}^{2}}{\left\{1-e^{-{\left[\alpha \left(1-\left[1+ax/\left(1+a\right)\right]e^{-ax}\right)\right]}^{2}}\right\}}^{\theta -1},\\ \; & \;x>0, \end{array}$$

and $h_{TBXL}\left(x;\alpha ,\theta ,a\;\right)=0$ for x≤0, respectively. Figure 5 displays some curves of the pdf and hrf for several parameter values.

Figure 5 exposes that the pdf of the TBXL distribution can be decreasing or unimodal and right-skewed. The hrf can decrease, increase, or be unimodal. As for the TBXE or TBXR distribution, the TBXL distribution dominates its parental distribution in terms of the diversity of pdf and hrf forms.

Based on Equation (6), the qf of the TBXL distribution is:

(11) $$\begin{array}{cc} \; & Q_{TBXL}\left(u;\alpha ,\theta ,a\right)=\\ \; & -\frac{1}{a}-1-\frac{1}{a}W_{-1}\left[\left(\frac{1}{\alpha }{\left\{-\log\left[1-u^{1/\theta }\left(1-e^{-\alpha^{2}}\right)\right]\right\}}^{1/2}-1\right)\left(a+1\right)e^{-1-a}\right],\\ \; & \;u\in (0,1), \end{array}$$

where $W_{-1}\left(x\right)$ refers to the “negative branch” of the standard Lambert function.

Thus, the quantile measures SK and KU can be expressed through Equations (7) and (8), respectively. A graphical work on these measures is proposed in Figure 4.

**Figure 6:**
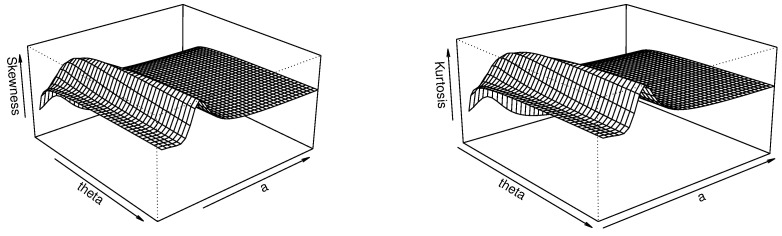
Three-dimensional plots of SK (**left**) and KU (**right**) of the TBXL distribution for a=1.

Figure 6 indicates that, for the fixed value of a=1, the skewness and kurtosis of TBXL are both increasing in θ and unimodal in *a*. Here again, these flexible properties are real assets for modeling perspectives.

To conclude this subsection, it should be mentioned that the TBXE, TBXL, and TBXR distributions are just three arbitrary examples of the TBX-G family, demonstrating sufficient qualities to be used in many statistical scenarios. In particular, the corresponding models are able to fit a plethora of skewed data to the right. Other interesting special distributions with different support can be analyzed in a similar way.

## 3. Mathematical Properties of the TBX-G Family

In this section, diverse mathematical properties of the TBX-G family are investigated. Whenever possible, the general results are applied at least to the TBXE distribution as described in Section 2.2.1.

### 3.1. Asymptotic Study

We now perform an asymptotic study of the main functions of the TBX-G family according to G(x;ω)→0 and G(x;ω)→1. When G(x;ω)→0, by using the equivalence of standard functions, we obtain:

$$\begin{array}{c} F_{TBX-G}\left(x;\alpha ,\theta ,\omega \right)∼\frac{\alpha^{2\theta }}{{\left(1-e^{-\alpha^{2}}\right)}^{\theta }}G{\left(x;\omega \right)}^{2\theta } \end{array}$$

and:

$$\begin{array}{c} f_{TBX-G}\left(x;\alpha ,\theta ,\omega \right)∼h_{TBX-G}\left(x;\alpha ,\theta ,\omega \right)∼\frac{2\theta \alpha^{2\theta }}{{\left(1-e^{-\alpha^{2}}\right)}^{\theta }}g\left(x;\omega \right)G{\left(x;\omega \right)}^{2\theta -1}. \end{array}$$

The parameter θ has a significant effect on the rate of convergence (or divergence), more than the parameter α, which only appears in the proportional constant.

When G(x;ω)→1, through the use of similar equivalence techniques, we have:

$$\begin{array}{c} F_{TBX-G}\left(x;\alpha ,\theta ,\omega \right)=1-\frac{2\alpha^{2}\theta }{e^{\alpha^{2}}-1}\bar{G}\left(x;\omega \right),\;f_{TBX-G}\left(x;\alpha ,\theta ,\omega \right)∼\frac{2\alpha^{2}\theta }{e^{\alpha^{2}}-1}g\left(x;\omega \right) \end{array}$$

and $h_{TBX-G}\left(x;\alpha ,\theta ,\omega \right)∼g\left(x;\omega \right)/\bar{G}\left(x;\omega \right)$, recalling that $\bar{G}\left(x;\omega \right)=1-G\left(x;\omega \right)$. This last equivalence function corresponds to the hrf of the reference distribution. Thus, in the case of G(x;ω)→1, we observe that the parameters α and θ only have a “proportional constant effect”. Furthermore, the above results show that the choice of the reference distribution is crucial in the asymptotic behavior of functions of the TBX-G family, among others.

If we apply these results to the TBXE distribution, the following equivalences are obtained. When x→0, we have:

$$\begin{array}{c} F_{TBXE}\left(x;\alpha ,\theta ,\beta \right)∼\frac{\alpha^{2\theta }}{{\left(1-e^{-\alpha^{2}}\right)}^{\theta }}\beta^{2\theta }x^{2\theta } \end{array}$$

and:

$$\begin{array}{c} f_{TBXE}\left(x;\alpha ,\theta ,\beta \right)∼h_{TBXE}\left(x;\alpha ,\theta ,\beta \right)∼\frac{2\theta \alpha^{2\theta }}{{\left(1-e^{-\alpha^{2}}\right)}^{\theta }}\beta^{2\theta }x^{2\theta -1}, \end{array}$$

showing a polynomial rate for this extreme limit, with a threshold parameter value at θ=1/2. On the other hand, when x→+∞, we have:

$$\begin{array}{c} F_{TBXE}\left(x;\alpha ,\theta ,\beta \right)=1-\frac{2\alpha^{2}\theta }{e^{\alpha^{2}}-1}e^{-\beta x},\;f_{TBXE}\left(x;\alpha ,\theta ,\beta \right)∼\frac{2\alpha^{2}\theta \beta }{e^{\alpha^{2}}-1}e^{-\beta x} \end{array}$$

and $h_{TBXE}\left(x;\alpha ,\theta ,\beta \right)∼\beta$. Thus, in all cases, $f_{TBXE}\left(x;\alpha ,\theta ,\beta \right)$ converges to zero with an exponential decay, whereas $h_{TBXE}\left(x;\alpha ,\theta ,\beta \right)$ tends to the constant β. These results are also useful for proving the existence of some integral quantities involving these functions, such as those related to Equation (4).

### 3.2. First-Order Stochastic Dominance Study

First-order stochastic dominance is a global concept that allows the comparison of several distributions. We refer the reader to [39]. The TBX-G family has a first-order stochastic dominance property, which is discussed in the following proposition.

**Proposition** **1.** *For $\alpha_{2}\geq \alpha_{1}$, $\theta_{2}\geq \theta_{1}$, and x∈R, we have:*

$$\begin{array}{c} F_{TBX-G}\left(x;\alpha_{1},\theta_{2},\omega \right)\leq F_{TBX-G}\left(x;\alpha_{2},\theta_{1},\omega \right). \end{array}$$

*That is, the TBX-G family with parameter vector $\left(x;\alpha_{1},\theta_{2},\omega \right)$ first-order stochastically dominates the TBX-G family with parameter vector $\left(x;\alpha_{2},\theta_{1},\omega \right)$.*

**Proof.**  First of all, note that we can write $F_{TBX-G}\left(x;\alpha ,\theta ,\omega \right)=v^{\theta }$ with:

$$\begin{array}{c} v=\left[1-e^{-{\left(\alpha G(x;\omega )\right)}^{2}}\right]/\left(1-e^{-\alpha^{2}}\right)\in \left[0,1\right]. \end{array}$$

Therefore, $F_{TBX-G}\left(x;\alpha ,\theta ,\omega \right)$ is obviously decreasing with respect to the parameter θ. On the other hand, after some developments, we obtain:

$$\begin{array}{c} \frac{\partial }{\partial \alpha }F_{TBX-G}\left(x;\alpha ,\theta ,\omega \right)=-2\alpha \theta F_{TBX-G}\left(x;\alpha ,\theta ,\omega \right)\frac{r[G(x;\omega )]}{\left(e^{\alpha^{2}}-1\right)\left(e^{\alpha^{2}G{\left(x;\omega \right)}^{2}}-1\right)}, \end{array}$$

where $r\left(x\right)=e^{\alpha^{2}x^{2}}-x^{2}e^{\alpha^{2}}+x^{2}-1$. Thus, only the sign of r(x) requires a particular work to determine the one of $\partial F_{TBX-G}\left(x;\alpha ,\theta ,\omega \right)/\partial \alpha$. The analysis of this function for x∈[0,1] gives: r(0)=r(1)=0, and that r(x) is decreasing over $\left[0,x_{\alpha }\right]$ and increasing over $\left[x_{\alpha },1\right]$ with $x_{\alpha }={\left\{\log\left[\left(e^{\alpha^{2}}-1\right)/\alpha^{2}\right]\right\}}^{1/2}/\alpha$. Consequently, r(x)≤0 for x∈[0,1], implying that $\partial F_{TBX-G}\left(x;\alpha ,\theta ,\omega \right)/\partial \alpha >0$. Hence, $F_{TBX-G}\left(x;\alpha ,\theta ,\omega \right)$ is increasing with respect to the parameter α. This concludes the proof.  □

Proposition 1 gives the full understanding of the role of the parameters α and θ on a certain hierarchy between the cdfs of the TBX-G family.

### 3.3. Series Expansion Study

The next result proposes a series expansion of the exponentiated pdf of the TBX-G family in terms of certain manageable functions derived from the reference distribution.

**Proposition** **2.** *Let p>0. Then, the following expansion holds:*

$$\begin{array}{cc} f_{TBX-G}{\left(x;\alpha ,\theta ,\omega \right)}^{p} & =\sum_{\left(k,\ell ,m\right)\in N^{3}}d_{k,\ell ,m}^{\left[p\right]}v_{G}\left(x;m,p,\omega \right), \end{array}$$

*where N={0,1,2…},*

(12) dk,ℓ,m[p]=(2θα2)p(1−e−α2)θpp(θ−1)k2ℓ+pm(−1)k+ℓ+m1ℓ!(k+p)ℓα2ℓ

*and:*

$$v_{G}\left(x;m,p,\omega \right)=g{\left(x;\omega \right)}^{p}\bar{G}{\left(x;\omega \right)}^{m}.$$

**Proof.**  Based on Equation (3), for any x∈R, it is clear that:

$$f_{TBX-G}{\left(x;\alpha ,\theta ,\omega \right)}^{p}=\frac{{\left(2\theta \alpha^{2}\right)}^{p}}{{\left(1-e^{-\alpha^{2}}\right)}^{\theta p}}g{\left(x;\omega \right)}^{p}G{\left(x;\omega \right)}^{p}e^{-p{\left(\alpha G(x;\omega )\right)}^{2}}{\left[1-e^{-{\left(\alpha G(x;\omega )\right)}^{2}}\right]}^{p(\theta -1)}.$$

It follows from the binomial theorem (generalized version), the series expansion of the exponential function, and the binomial theorem (generalized version) that:

fTBX−G(x;α,θ,ω)p=(2θα2)p(1−e−α2)θpg(x;ω)pG(x;ω)p∑k∈Np(θ−1)k(−1)ke−(k+p)αG(x;ω)2=(2θα2)p(1−e−α2)θpg(x;ω)p∑k∈Np(θ−1)k(−1)k∑ℓ∈N1ℓ!(−1)ℓ(k+p)ℓα2ℓG(x;ω)2ℓ+p=(2θα2)p(1−e−α2)θpg(x;ω)p∑k∈Np(θ−1)k(−1)k∑ℓ∈N1ℓ!(−1)ℓ(k+p)ℓα2ℓ∑m∈N2ℓ+pm(−1)mG¯(x;ω)m=∑(k,ℓ,m)∈N3(2θα2)p(1−e−α2)θpp(θ−1)k2ℓ+pm(−1)k+ℓ+m1ℓ!(k+p)ℓα2ℓg(x;ω)pG¯(x;ω)m=∑(k,ℓ,m)∈N3dk,ℓ,m[p]vG(x;m,p,ω).

Proposition 2 is proven. □

By tuning the values of *p*, we can apply Proposition 2 to obtain series expansions of well-known measures and functions. In particular, by taking p=1, the pdf of the TBX-G family can be expanded as:

$$\begin{array}{cc} f_{TBX-G}\left(x;\alpha ,\theta ,\omega \right) & =\sum_{\left(k,\ell ,m\right)\in N^{3}}d_{k,\ell ,m}^{\left[1\right]}v_{G}\left(x;m,1,\omega \right), \end{array}$$

where dk,ℓ,m[1]=2θα2θ−1k2ℓ+1m(−1)k+ℓ+m(k+1)ℓα2ℓ/[(1−e−α2)θℓ!] and $v_{G}\left(x;m,1,\omega \right)=g\left(x;\omega \right)\bar{G}{\left(x;\omega \right)}^{m}$. In full generality, the function $v_{G}\left(x;m,p,\omega \right)$ is simple and remains quite manageable for most of the standard reference distributions. In particular, in the framework of the:

- TBXE distribution, for x>0, we have:
  $$v_{G}\left(x;m,p,\omega \right)=g_{E}{\left(x;\omega \right)}^{p}{\bar{G}}_{E}{\left(x;\omega \right)}^{m}=\beta^{p}e^{-(p+m)\beta x};$$
- TBXR distribution, for x>0, we have:
  $$v_{G}\left(x;m,p,\omega \right)=g_{R}{\left(x;\omega \right)}^{p}{\bar{G}}_{R}{\left(x;\omega \right)}^{m}=\rho^{p}x^{p}e^{-\left(p+m\right)\rho x^{2}/2};$$
- TBXL distribution, for x>0, we have:
  vG(x;m,p,ω)=gL(x;ω)pG¯L(x;ω)m=a2p(1+a)p(1+x)p1+ax1+ame−(p+m)ax=a2p(1+a)m+p∑k=0mmkak(1+x)k+pe−(p+m)ax.

The three functions above are linear combinations of polynomial-exponential functions, which are involved in many well-mastered integral formulas.

### 3.4. Tsallis Entropy Study

In order to study the “random uncertainty” behind the TBX-G family, we propose to investigate the Tsallis entropy measure. Further details on this classical entropy measure can be found in [40,41]. Basically, the Tsallis entropy measure of the TBX-G family at *p* with p>0 and p≠1 is defined as:

$$\begin{array}{cc} T_{p} & =\frac{1}{p-1}\left[1-\int_{R}f_{TBX-G}{\left(x;\alpha ,\theta ,\omega \right)}^{p}dx\right]\\ \; & =\frac{1}{p-1}\left[1-\frac{{\left(2\theta \alpha^{2}\right)}^{p}}{{\left(1-e^{-\alpha^{2}}\right)}^{\theta p}}\int_{R}g{\left(x;\omega \right)}^{p}G{\left(x;\omega \right)}^{p}e^{-p{\left(\alpha G(x;\omega )\right)}^{2}}{\left[1-e^{-{\left(\alpha G(x;\omega )\right)}^{2}}\right]}^{p(\theta -1)}dx\right]. \end{array}$$

For most of the referenced parental distributions, this integral is not easy to determine. The integral term can be evaluated numerically by any integral approximation procedure. An expression involving sums of coefficients can be derived from Proposition 2. Indeed, by applying this proposition directly, subject to the mathematical correctness of the exchange of the signs ∑ and ∫, we have:

(13) $$\begin{array}{c} \int_{R}f_{TBX-G}{\left(x;\alpha ,\theta ,\omega \right)}^{p}dx=\sum_{\left(k,\ell ,m\right)\in N^{3}}d_{k,\ell ,m}^{\left[p\right]}\phi_{m}^{\left[p\right]}, \end{array}$$

where dk,ℓ,m[p]=(2θα2)pp(θ−1)k2ℓ+pm(−1)k+ℓ+m(k+p)ℓα2ℓ/[ℓ!(1−e−α2)θp] and $\phi_{m}^{\left[p\right]}=\int_{R}v_{G}\left(x;m,p,\omega \right)dx$. Therefore, the Tsallis entropy measure of the TBX-G family at *p* can be expressed as:

$$\begin{array}{cc} T_{p} & =\frac{1}{p-1}\left[1-\sum_{\left(k,\ell ,m\right)\in N^{3}}d_{k,\ell ,m}^{\left[p\right]}\phi_{m}^{\left[p\right]}\right]. \end{array}$$

A simple approximation can be deduced; we have $T_{p}\approx \left[1-\sum_{\left(k,\ell ,m\right)=0_{3}}^{50_{3}}d_{k,\ell ,m}^{\left[p\right]}\phi_{m}^{\left[p\right]}\right]/\left(p-1\right)$, the upper limit of 50 being chosen arbitrarily large. This expansion remains manageable for most of the referenced distributions. For instance, in the context of the TBXE distribution, provided to (2θ−1)p>−1, it can be applied with:

$$\phi_{m}^{\left[p\right]}=\int_{R}v_{G}\left(x;m,p,\omega \right)dx=\beta^{p}\int_{\left(0,+\infty \right)}e^{-(p+m)\beta x}dx=\frac{\beta^{p-1}}{p+m}.$$

The computation of $T_{p}$ becomes simple.

### 3.5. Moment Study

The TBX-G family is now subjected to moment analysis. We start with a random variable *X* whose distribution belongs to the TBX-G family. Then, based on Equation (4), upon the existence condition, the *s*-th order moment (about the origin) of *X* is:

$$\begin{array}{cc} \mu_{s}^{\prime } & =E\left(X^{s}\right)=\int_{R}x^{s}f_{TBX-G}\left(x;\alpha ,\theta ,\omega \right)dx\\ \; & =\frac{2\theta \alpha^{2}}{{\left(1-e^{-\alpha^{2}}\right)}^{\theta }}\int_{R}x^{s}g\left(x;\omega \right)G\left(x;\omega \right)e^{-{\left(\alpha G(x;\omega )\right)}^{2}}{\left[1-e^{-{\left(\alpha G(x;\omega )\right)}^{2}}\right]}^{\theta -1}dx. \end{array}$$

Another integral expression is given by the use of Equation (6); we have:

$$\begin{array}{cc} \mu_{s}^{\prime } & =\int_{\left(0,1\right)}{\left[Q_{TBX-G}\left(u;\alpha ,\theta ,\omega \right)\right]}^{s}du\\ \; & =\int_{\left(0,1\right)}{\left[Q_{G}\left(\frac{1}{\alpha }{\left\{-\log\left[1-u^{1/\theta }\left(1-e^{-\alpha^{2}}\right)\right]\right\}}^{1/2},\omega \right)\right]}^{s}du. \end{array}$$

As for the integral expression of the Tsallis entropy measure, for most of the reference parental distributions, this integral is not simply expressible. A numerical approach is always possible to obtain a suitable evaluation. A series expansion approach can be derived from Proposition 2. By applying this result to p=1, subject to the mathematical correctness of the exchanges of the signs ∑ and ∫, we have:

(14) $$\begin{array}{c} \mu_{s}^{\prime }=\sum_{\left(k,\ell ,m\right)\in N^{3}}d_{k,\ell ,m}^{\left[1\right]}\xi_{m}^{\left[s\right]}, \end{array}$$

where dk,ℓ,m[1]=2θα2θ−1k2ℓ+1m(−1)k+ℓ+m(k+1)ℓα2ℓ/[ℓ!(1−e−α2)θ] and:

$$\xi_{m}^{\left[s\right]}=\int_{R}x^{s}v_{G}\left(x;m,1,\omega \right)dx.$$

Based on this expansion, we conjecture the following approximation:

$$\mu_{s}^{\prime }\approx \sum_{\left(k,\ell ,m\right)=0_{3}}^{50_{3}}d_{k,\ell ,m}^{\left[1\right]}\xi_{m}^{\left[s\right]},$$

which remains quite manageable as a finite sum of given coefficients. As an example of application, within the framework of the TBXE distribution, the previous sums can be applied with:

$$\begin{array}{c} \xi_{m}^{\left[s\right]}=\int_{\left(0,+\infty \right)}x^{s}\beta e^{-(m+1)\beta x}dx=\frac{\Gamma (s+1)}{\beta^{s}}\frac{1}{{\left(m+1\right)}^{s+1}}, \end{array}$$

where Γ(x) refers to the standard gamma function.

Similarly, the *s*-th order incomplete moment of *X* at the threshold value *t* is defined by:

$$\begin{array}{cc} \mu_{s}^{\prime }\left(t\right) & =E\left(X^{s}I\left(X\leq t\right)\right)=\int_{\left(-\infty ,t\right)}x^{s}f_{TBX-G}\left(x;\alpha ,\theta ,\omega \right)dx\\ \; & =\frac{2\theta \alpha^{2}}{{\left(1-e^{-\alpha^{2}}\right)}^{\theta }}\int_{\left(-\infty ,t\right)}x^{s}g\left(x;\omega \right)G\left(x;\omega \right)e^{-{\left(\alpha G(x;\omega )\right)}^{2}}{\left[1-e^{-{\left(\alpha G(x;\omega )\right)}^{2}}\right]}^{\theta -1}dx. \end{array}$$

In the same spirit as Equation (14), an expansion of $\mu_{s}^{\prime }\left(t\right)$ is given by:

(15) $$\begin{array}{c} \mu_{s}^{\prime }\left(t\right)=\sum_{\left(k,\ell ,m\right)\in N^{3}}d_{k,\ell ,m}^{\left[1\right]}\xi_{m}^{\left[s\right]}\left(t\right), \end{array}$$

where $\xi_{m}^{\left[s\right]}\left(t\right)=\int_{\left(-\infty ,t\right)}x^{s}v_{G}\left(x;m,1,\omega \right)dx$. In the context of the TBXE distribution, we simply have:

$$\begin{array}{c} \xi_{m}^{\left[s\right]}\left(t\right)=\int_{\left(0,t\right)}x^{s}\beta e^{-(m+1)\beta x}dx=\frac{1}{\beta^{s}}\frac{\gamma (s+1,(m+1)\beta t)}{{\left(m+1\right)}^{s+1}}, \end{array}$$

where γ(a,x) refers to the standard lower incomplete gamma function. Applications of incomplete moments can be found in [2]. Next, we will use them to present some useful risk measures.

### 3.6. Risk Measures

We now focus on some risk measures of interest defined by the TBX-G family. In full generality, the most commonly used as a standard financial market risk measure is the value at risk. It is also known as the quantile risk measure or the quantile premium principle. It always depends on a certain degree of confidence, denoted by *q*. Mathematically, it corresponds to the *q*-th quantile of the considered distribution. For the TBX-G family, based on Equation (6), it is directly expressed as:

(16) $$VaR_{q}=Q_{TBX-G}\left(q;\alpha ,\theta ,\omega \right)=Q_{G}\left(\frac{1}{\alpha }{\left\{-\log\left[1-q^{1/\theta }\left(1-e^{-\alpha^{2}}\right)\right]\right\}}^{1/2},\omega \right),\;q\in \left(0,1\right).$$

It can be easily explicated for the TBXE, TBXR, and TBXL distributions through Equations (9), (10), and (11), respectively. Another important financial risk measure is the expected shortfall, which is often considered as a better measure than value at risk. It is defined by the following expression:

(17) $$\begin{array}{cc} ES_{q} & =\frac{1}{q}\int_{\left(0,q\right)}VaR_{x}dx\\ \; & =\frac{1}{q}\int_{\left(0,q\right)}Q_{G}\left(\frac{1}{\alpha }{\left\{-\log\left[1-x^{1/\theta }\left(1-e^{-\alpha^{2}}\right)\right]\right\}}^{1/2},\omega \right)dx,\;q\in \left(0,1\right). \end{array}$$

**Figure 7:**
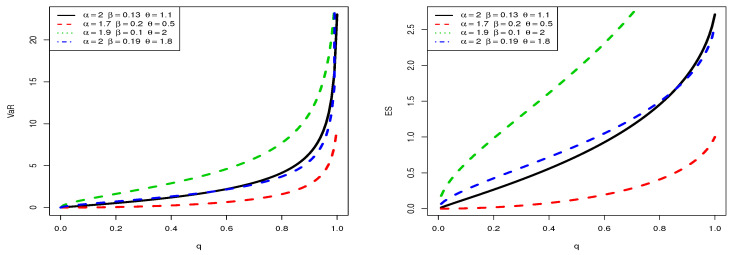
Plots of $VaR_{q}$ (**left**) and $ES_{q}$ (**right**) of the TBXE distribution for some parameter values.

For any parental distribution, it can be determined at least numerically. As an application, let us investigate the measures $VaR_{q}$ and $ES_{q}$ related to the TBXE distribution. For selected values of the parameters, the changes in the curves of $VaR_{q}$ and $ES_{q}$ are illustrated in Figure 7.

From Figure 7, it is observed that $VaR_{q}$ is exclusively convex with respect to *q* and shows various “elbow degrees”, whereas $ES_{q}$ can be either concave or convex, according to the values of the parameters. This indicates a certain flexibility in these risk measures.

To be complete, one can also mention some useful related tail measures, such as the tail value at risk, tail variance, and tail variance premium defined by $TVaR_{q}=\left[\mu_{1}^{\prime }-\mu_{1}^{\prime }\left(VaR_{q}\right)\right]/\left(1-q\right)$, $TV_{q}\left(x\right)=\left[\mu_{2}^{\prime }-\mu_{2}^{\prime }\left(VaR_{q}\right)\right]/\left(1-q\right)-{\left[TVaR_{q}\right]}^{2}$, and $TVP_{q}\left(X\right)=TVaR_{q}$$+\;\delta TV_{q}$ for δ∈(0,1), respectively. All these measures can be studied numerically. Furthermore, the series expansions determined in Equations (14) and (15) can be used to provide sum approximations of these risk measures. We refer the reader to [42,43] for a detailed description of them.

## 4. Statistical and Inferential Approaches

The main purpose of the TBX-G family is to provide statistical models useful for analyzing different types of data. The first step in this direction is the accurate estimation of the related parameters. Here, the ML technique is employed.

### 4.1. Methodology

In order to introduce the ML technique adapted to the TBX-G family, let us consider *n* realizations $x_{1},\ldots ,x_{n}$ of *n* independent and identically random variables with the cdf given by Equation (2). Then, the maximum likelihood estimate (MLE) of the parameter vector (α,θ,ω) is given as $\left(\hat{\alpha },\hat{\theta },\hat{\omega }\right)={\mathrm{argmax}}_{(\alpha ,\theta ,\omega )\in D}L\left(\alpha ,\theta ,\omega \right)$, where *D* denotes the domain of the parameters and L(α,θ,ω) denotes the likelihood function defined by:

$$\begin{array}{cc} L(\alpha ,\theta ,\omega ) & =\prod_{i=1}^{n}f_{TBX-G}\left(x_{i};\alpha ,\theta ,\omega \right)\\ \; & =\prod_{i=1}^{n}\left[\frac{2\theta \alpha^{2}}{{\left(1-e^{-\alpha^{2}}\right)}^{\theta }}g\left(x_{i};\omega \right)G\left(x_{i};\omega \right)e^{-{\left(\alpha G(x_{i};\omega )\right)}^{2}}{\left[1-e^{-{\left(\alpha G(x_{i};\omega )\right)}^{2}}\right]}^{\theta -1}\right]. \end{array}$$

Note that the components of the MLE $\left(\hat{\alpha },\hat{\theta },\hat{\omega }\right)$ are also called the MLEs of the corresponding component of (α,θ,ω). Subject to mathematical correctness, one can find the MLEs through the maximization of the log-likelihood function ℓ(α,θ,ω)=logL(α,θ,ω), which can be expressed as:

$$\begin{array}{cc} \ell (\alpha ,\theta ,\omega ) & =n\log\left(2\theta \right)+2n\log\left(\alpha \right)-n\theta \log\left(1-e^{-\alpha^{2}}\right)+\sum_{i=1}^{n}\log g\left(x_{i};\omega \right)\\ \; & +\sum_{i=1}^{n}\log G\left(x_{i};\omega \right)-\alpha^{2}\sum_{i=1}^{n}G{\left(x_{i};\omega \right)}^{2}+\left(\theta -1\right)\sum_{i=1}^{n}\log\left[1-e^{-{\left(\alpha G(x_{i};\omega )\right)}^{2}}\right]. \end{array}$$

The desired MLEs can be derived in a theoretical way by manipulating the components of the score vector $\left(S_{\alpha },S_{\theta },S_{\omega }\right)$, where:

$$\begin{array}{cc} S_{\alpha } & =\frac{\partial }{\partial \alpha }\ell \left(\alpha ,\theta ,\omega \right)=\frac{2n}{\alpha }-\frac{2\alpha n\theta e^{-\alpha^{2}}}{1-e^{-\alpha^{2}}}-2\alpha \sum_{i=1}^{n}G{\left(x_{i};\omega \right)}^{2}\\ \; & +2\alpha \left(\theta -1\right)\sum_{i=1}^{n}\frac{G{\left(x_{i};\omega \right)}^{2}e^{-{\left(\alpha G(x_{i};\omega )\right)}^{2}}}{1-e^{-{\left(\alpha G(x_{i};\omega )\right)}^{2}}}, \end{array}$$

$$\begin{array}{cc} S_{\theta } & =\frac{\partial }{\partial \theta }\ell \left(\alpha ,\theta ,\omega \right)=\frac{n}{\theta }-n\log\left(1-e^{-\alpha^{2}}\right)+\sum_{i=1}^{n}\log\left[1-e^{-{\left(\alpha G(x_{i};\omega )\right)}^{2}}\right] \end{array}$$

and, by denoting $\omega =(\omega_{1},\ldots ,\omega_{p})$, $S_{\omega }=\left(S_{\omega_{1}},\ldots ,S_{\omega_{p}}\right)$ with, for j=1,…,p,

$$\begin{array}{cc} S_{\omega_{j}} & =\frac{\partial }{\omega_{j}}\ell \left(\alpha ,\theta ,\omega \right)=\sum_{i=1}^{n}\frac{g_{j}^{\prime }\left(x_{i};\omega \right)}{g(x_{i};\omega )}+\sum_{i=1}^{n}\frac{G_{j}^{\prime }\left(x_{i};\omega \right)}{G(x_{i};\omega )}-2\alpha^{2}\sum_{i=1}^{n}G\left(x_{i};\omega \right)G_{j}^{\prime }\left(x_{i};\omega \right)\\ \; & +2\alpha^{2}\left(\theta -1\right)\sum_{i=1}^{n}\frac{G\left(x_{i};\omega \right)G_{j}^{\prime }\left(x_{i};\omega \right)e^{-{\left(\alpha G(x_{i};\omega )\right)}^{2}}}{1-e^{-{\left(\alpha G(x_{i};\omega )\right)}^{2}}}, \end{array}$$

with $g_{j}^{\prime }\left(x_{i};\omega \right)=\partial g\left(x_{i};\omega \right)/\partial \omega_{j}$ and $G_{j}^{\prime }\left(x_{i};\omega \right)=\partial G\left(x_{i};\omega \right)/\partial \omega_{j}$. By solving the equations of the following system: $\left(S_{\alpha },S_{\theta },S_{\omega }\right)=0_{p+2}$ simultaneously, we obtain $\left(\hat{\alpha },\hat{\theta },\hat{\omega }\right)$. In general, these equations cannot be solved analytically, so statistical software is necessary to approximate them in a precise way. Furthermore, thanks to the knowledge of the ML theory, we can determine the standard errors (SEs) of the MLEs, as well as confidence intervals or likelihood ratio tests for the parameters. Further details on the ML technique are available in [44]. Last but not least, based on the MLEs $\left(\hat{\alpha },\hat{\theta },\hat{\omega }\right)$, natural estimates for the pdf and cdf of the TBX-G family are expressed as $f_{TBX-G}\left(x;\hat{\alpha },\hat{\theta },\hat{\omega }\right)$ and $F_{TBX-G}\left(x;\hat{\alpha },\hat{\theta },\hat{\omega }\right)$, respectively. Other unknown measures and functions can be estimated by the same substitution technique.

### 4.2. Simulation

Here, we provide a numerical complement to the above theory by performing a simulation study on the behavior of the ML technique. Only the TBXE model was considered. To this aim, for some selected parameter values, we generated 10,000 samples from the TBXE distribution, each of sample size n=30+50k with k=0,1,2,3,4,5. The assessment was based on standard benchmark measures, such as the average estimates (AEs) of the estimates and the root mean squared errors (RMSEs). The R-Statistical Computing Environment was used (see [45]). The results of our simulation study are given in Table 1.

From Table 1, we see that the ML technique shows consistency and is quite satisfactory; AEs converge to expected values as the sample size increases, and RMSEs decrease as the sample size increases.

## 5. Applications to Actuarial and Financial Data

In this section, the fit power of the TBXE distribution is revealed through the analysis of two different datasets. Furthermore, based on these data, risk measures are estimated.

### 5.1. Data Fitting

The first dataset, labeled as D1, is a right-skewed real dataset from the insurance field. It represents monthly measures of unemployment insurance from July 2008 to April 2013 reported by the Maryland Department of Labor. The data consists of 21 variables, and we analyzed, in particular, Variable Number 12. The data are available at the following uniform resource locator: https://catalog.data.gov/dataset/unemployment-insurance-data-july-2008-to-april-2013 (accessed on 23 May 2021).

The second dataset, labeled as D2, includes actual monthly tax revenues in Egypt from January 2006 to November 2010. The related reference for these data is [46].

Our methodology aims to compare the goodness-of-fit results and certain measures of discrimination of the TBXE model with those of other well-known competing models, including the BX, exponentiated exponential (EE), exponential (E), Marshall–Olkin exponential (MOE), exponentiated Weibull (EW), odd Weibull exponential (OWE), Weibull (W), and Topp–Leone exponential (TLE) models.

To achieve this objective, the AdequacyModel package of the R-Statistical Computing Environment was used. For each dataset, we provide the MLEs and the SEs of the MLEs of the proposed and other competitive models. The log-likelihood function was calculated at the obtained MLEs ($\hat{\ell }$). Some well-established goodness-of-fit statistics such as the Akaike information criterion (AIC), consistent AIC (CAIC), Bayesian information criterion (BIC), Hannan–Quinn information criterion (HQIC), Anderson–Darling ($A^{*}$), Cramér–von Mises ($W^{*}$), and Kolmogorov–Smirnov (K.S) were considered for model comparison purposes. Low values of the goodness-of-fit statistics and high K-S *p*-values indicate good fits.

MLEs and their respective SEs for the TBXE, BX, EE, E, MOE, EW, OWE, W and TLE models, which were fit to D1 and D2, are reported in Table 2.

The values of the goodness-of-fit statistics are reported in Table 3 and Table 4 for D1 and D2, respectively.

From Table 3 and Table 4, we can observe that the TBXE model gives a better fit to D1 and D2 compared to all the other competing models, since it has the minimum values of AIC, CAIC, BIC, HQIC, $A^{*}$, $W^{*}$, and K.S and the maximum values of K.S *p*-values. Despite its relative classicism, we can consider that the EE model is the second best model.

**Figure 8:**
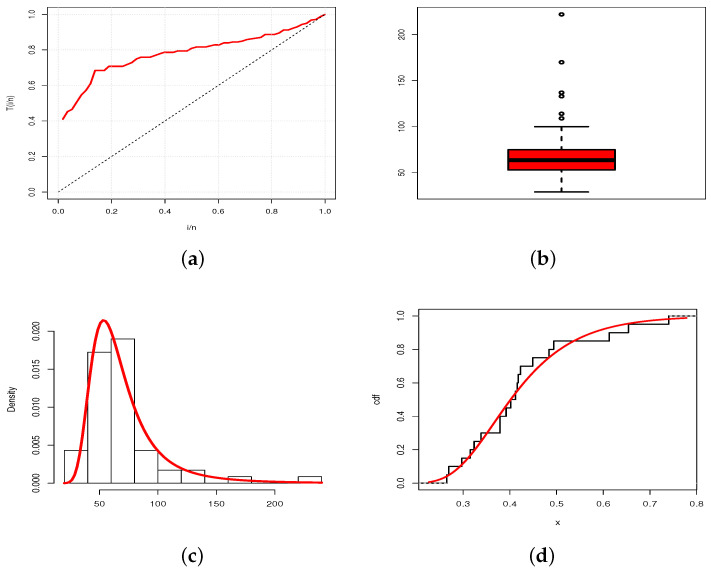
(**a**) TTT plot; (**b**) box plot; (**c**) plot of the estimated pdf over the histogram; (**d**) plot of the estimated cdf over the empirical cdf for the TBXE model for D1.

**Figure 9:**
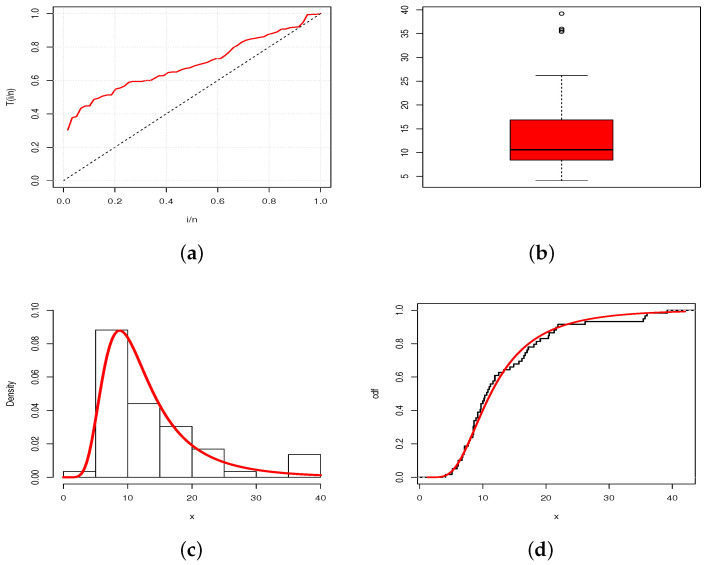
(**a**) TTT plot; (**b**) box plot; (**c**) plot of the estimated pdf over the histogram; (**d**) plot of the estimated cdf over the empirical cdf for the TBXE model for D2.

A visual approach is given in Figure 8 and Figure 9, where the total time on test (TTT) plots and box plots of the data are presented, as well as the estimated pdfs of the TBXE model over the histograms of the data and estimated cdfs of the TBXE model over the empirical cdfs of the data, for D1 and D2, respectively.

From Figure 8 and Figure 9, we see that the estimated pdfs and cdfs fit the corresponding empirical objects quite well, despite two different levels of right skewness in the data and the presence of outliers.

### 5.2. Estimation of $VaR_{q}$ and $ES_{q}$

Here, we investigate the estimation of the risk measures $VaR_{q}$ and $ES_{q}$ for the TBXE model based on D1 and D2. The considered estimates were based on the mathematical expression of $VaR_{q}$ and $ES_{q}$ where the parameters were replaced by their MLEs. We carried out a comparative study of these estimated risk measures for the TBXE model with their homologous models, namely the BX, EE, EW, W, and E models. It should be noted that a model with higher values of the considered risk measures is supposed to have a heavier tail. Table 5 and Table 6 provide the estimates of $VaR_{q}$ and $ES_{q}$ for D1, respectively, and Table 7 and Table 8 give the estimates of $VaR_{q}$ and $ES_{q}$ for D2, respectively.

**Figure 10:**
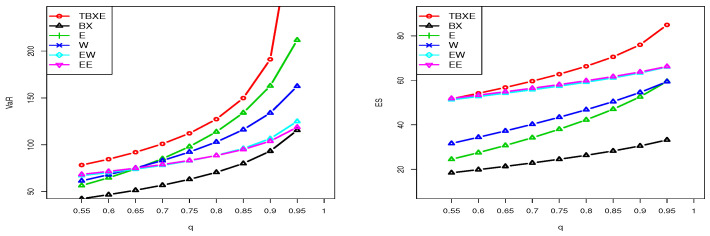
Plots of the estimated $VaR_{q}$ (**left**) and estimated $ES_{q}$ (**right**) of the considered models for D1.

**Figure 11:**
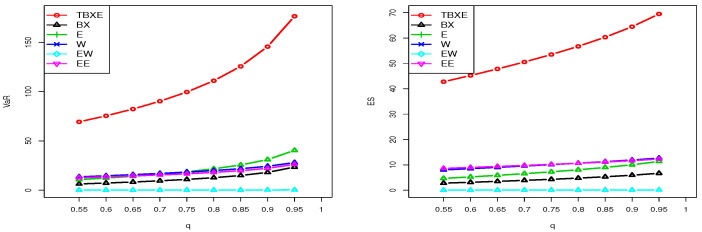
Plots of the estimated $VaR_{q}$ (**left**) and estimated $ES_{q}$ (**right**) of the considered models for D2.

The results in Table 5, Table 6, Table 7 and Table 8 show that the TBXE model has higher values for both risk measures compared to their counterparts for the BX, EE, EW, W, and E models. This observation is clearly visualized in Figure 10 and Figure 11 for D1 and D2, respectively.

The graphical demonstration of the models in Figure 10 and Figure 11 also reveals that the proposed model is heavier than the BX, EE, EW, W, and E models. See [43,47] for a detailed discussion of $VaR_{q}$ and $ES_{q}$ and their calculation using the R-Statistical Computing Environment.

## 6. Concluding Notes and Perspectives

### 6.1. Concluding Notes

In this article, we showed how the use of the “truncated-composed” scheme applied to the BX distribution can lead to new flexible statistical models for the analysis of right-skewed data. Precisely, we proceeded as follows. First, we defined the TBX-G family of distributions, discussed the motivations behind it, and studied its main properties of interest. Three particular examples of three-parameter family-owned distributions were presented in depth, with an emphasis on the one having the exponential distribution as a parent. The TBXE distribution was thus introduced and studied, revealing flexible decreasing and unimodal shapes and various asymmetric trends on the right concerning its pdf. Thanks to the ML technique applied to the model parameters, we demonstrated the power of the adjustment of the TBXE model through a solid simulation study and the analysis of two different and right-skewed datasets taken from the fields of actuarial science and finance. In addition, we showed that the TBXE model may be a better alternative to other famous survival models from the literature. Our results provide theoretical and practical assurance of the accuracy of the TBXE model for various statistical purposes, as well as the TBX-G family.

### 6.2. Perspectives

Other lines of research include the use of parental distributions with support on the real line, such as normal, Laplace, and logistic distributions, the development of various regression models, as well as the study of the multivariate version of the TBX-G family for the treatment of multivariate data. Naturally, thanks to their modeling characteristics, the distributions of the TBX-G family have potential applications in areas other than actuarial science and finance. We would like to mention the insurance industry, where there is a need for such flexible statistical models. In this regard, we may refer to the works in [48,49,50]. On the other hand, the proposed distributions may be useful in modeling tribo-fatigue systems and new materials. In particular, they can be involved in (i) a method of experimental study of friction in an active system, (ii) the state of volumetric damage of a tribo-fatigue system, (iii) the spatial stress–strain state of a tribo-fatigue system in the roll–shaft contact zone, (iv) modeling of the damaged state by the finite-element method on the simultaneous action of contact and noncontact loads, (v) the tribo-fatigue behavior of austempered ductile iron monica as a new structural material for a rail–wheel system, (vi) research on the tensile behavior of the new structural material monica, and (vii) measurement and real-time analysis of local damage in wear-and-fatigue tests. In this regard, we may refer the reader to [51,52,53].

## Figures and Tables

**Table 1:** Actual values, AEs, and RMSEs of the simulated data from the TBXE distribution for some parameter values.

Sample Size	Actual Values			AE			RMSE		
n	α	θ	β	$\hat{\alpha }$	$\hat{\theta }$	$\hat{\beta }$	$\hat{\alpha }$	$\hat{\theta }$	$\hat{\beta }$
30	0.3	0.7	1.2	0.5537	0.8124	1.1760	0.6695	0.2679	0.3346
80				0.5279	0.7408	1.1247	0.6429	0.1302	0.2472
130				0.4911	0.7296	1.1310	0.6031	0.0989	0.2173
180				0.4759	0.7232	1.1310	0.5765	0.0818	0.1976
230				0.4587	0.7161	1.1448	0.5442	0.0721	0.1795
280				0.4276	0.7161	1.1448	0.5144	0.0642	0.1662
30	1.3	2.7	0.6	1.0669	3.4192	0.6530	0.7543	4.7441	0.1966
80				1.1540	2.7689	0.6120	0.6604	0.8594	0.1576
130				1.1975	2.6818	0.6018	0.5846	0.6084	0.1398
180				1.2315	2.6669	0.5973	0.5241	0.5153	0.1284
230				1.2481	2.6672	0.5971	0.4783	0.4576	0.1203
280				1.2640	2.6628	0.5944	0.4472	0.4174	0.1134
30	0.8	0.8	0.9	0.6695	0.9178	0.9476	0.6962	0.3263	0.2809
80				0.7074	0.8290	0.8894	0.6706	0.1497	0.2144
130				0.7187	0.8125	0.8125	0.6477	0.1135	0.1934
180				0.7339	0.8066	0.8760	0.6190	0.0952	0.1819
230				0.7268	0.8042	0.8802	0.5901	0.0846	0.1694
280				0.7380	0.8020	0.8779	0.5683	0.0761	0.1610
30	1.5	0.9	0.9	0.9432	1.0338	1.2239	0.7431	0.3945	0.4349
80				1.1262	0.9287	1.0851	0.6901	0.1767	0.3340
130				1.2224	0.9121	1.0370	0.6314	0.1347	0.3021
180				1.2828	0.9060	1.0103	0.5801	0.1152	0.2839
230				1.3237	0.8999	0.9885	0.5365	0.1001	0.2634
280				1.3556	0.8984	0.9749	0.5114	0.0924	0.2560
30	1.2	0.8	0.6	0.7988	0.8992	0.7166	0.7192	0.3198	0.2379
80				0.9468	0.8176	0.6501	0.6862	0.1493	0.1816
130				1.0275	0.8052	0.6271	0.6413	0.1133	0.1653
180				1.0539	0.7964	0.6208	0.6045	0.0944	0.1535
230				1.0804	0.7953	0.6154	0.5765	0.0847	0.1478
280				1.0974	0.7944	0.6129	0.5420	0.0766	0.1401
30	1.0	1.0	0.8	0.7735	1.1469	0.8711	0.7156	0.4412	0.2654
80				0.8561	1.0253	0.8068	0.6855	0.1976	0.2076
130				0.8889	1.0075	0.7974	0.6465	0.1514	0.1881
180				0.9068	1.0008	0.7930	0.6066	0.1279	0.1739
230				0.9156	0.9984	0.7931	0.5716	0.1137	0.1634
280				0.9347	0.9945	0.7885	0.5448	0.1036	0.1553
30	1.2	0.8	0.9	0.8092	0.9033	1.0767	0.7121	0.3203	0.3530
80				0.9476	0.8189	0.9739	0.6910	0.1504	0.2734
130				1.0226	0.8027	0.9405	0.6481	0.1143	0.2457
180				1.0763	0.7996	0.9251	0.6020	0.0951	0.2319
230				1.0842	0.7964	0.9221	0.5752	0.0844	0.2212
280				1.1167	0.7933	0.9107	0.5486	0.0764	0.2123
30	0.5	1.8	1.5	0.9069	2.3930	1.4504	0.7638	1.4810	0.3880
80				0.6344	2.0147	1.4201	0.7482	0.5264	0.2887
130				0.5924	1.9439	1.4294	0.6190	0.3896	0.2482
180				0.5499	1.9137	1.4450	0.5578	0.3264	0.2088
230				0.5349	1.8937	1.4489	0.5239	0.2821	0.1848
280				0.5096	1.8782	1.4604	0.4914	0.2556	0.1630

**Table 2:** MLEs and SEs of the model parameters for D1 and D2, respectively.

Model	Parameters	MLEs (D1)	SEs (D1)	MLEs (D2)	SEs (D2)
TBXE	α	2.6510	0.282	1.8533	0.3379
	θ	10.4527	4.99	5.1464	2.0897
	β	0.0152	0.0040	0.1191	0.0356
BX	α	0.0200	0.0012	0.0644	0.0056
	θ	1.9912	0.4252	1.0310	0.1844
EE	α	0.05	0.006	0.1786	0.0232
	β	16.08	5.251	5.5321	1.4350
E	β	0.0142	0.0020	0.0741	0.0096
MOE	α	0.0664	0.0082	0.2092	0.0308
	a	72.1333	41.0232	11.5647	5.2019
EW	α	0.4333	0.4872	1.5481	0.9126
	β	0.6043	0.1992	0.4706	0.1308
	a	130.3842	219.0512	88.6904	8.4074
OWE	α	0.0028	0.0005	0.0164	0.0185
	a	14.2800	7.2812	6.6161	5.4439
	b	1.9155	0.1843	1.5472	1.5625
W	α	0.0029	0.0006	0.0069	0.0028
	β	1.3611	0.0552	1.8215	0.1339
TLE	α	0.0242	0.0028	0.0893	0.0116
	a	15.9758	5.1955	5.5322	1.4347

**Table 3:** Goodness-of-fit statistics and K.S *p*-value for D1.

Model	$-\hat{\ell }$	AIC	CAIC	BIC	HQIC	*A* ${}^{*}$	*W* ${}^{*}$	K.S	*p*-Value
TBXE	265.329	536.658	537.102	542.839	539.066	0.716	0.125	0.111	0.471
BX	275.364	554.728	554.947	558.849	556.334	2.416	0.444	0.182	0.044
EE	267.487	538.973	539.191	543.094	540.578	1.090	0.201	0.113	0.447
E	304.967	611.934	612.006	613.995	612.737	1.755	0.324	0.387	0.00001
MOE	274.318	552.636	552.854	556.757	554.241	2.218	0.400	0.140	0.204
EW	266.190	538.379	538.824	544.561	540.787	0.896	0.163	0.117	0.408
OWE	281.963	569.927	570.371	576.108	572.334	3.312	0.608	0.187	0.034
W	291.235	586.470	586.688	590.591	588.075	2.065	0.380	0.332	0.00001
TLE	267.487	538.973	539.191	543.094	540.578	1.091	0.202	0.113	0.445

**Table 4:** Goodness-of-fit statistics and K.S *p*-value for D2.

Model	$-\hat{\ell }$	AIC	CAIC	BIC	HQIC	*A* ${}^{*}$	*W* ${}^{*}$	K.S	*p*-Value
TBXE	265.3401	383.0907	383.5271	389.3233	385.5237	0.3621	0.0623	0.0703	0.9321
BX	275.3641	399.3927	399.6070	403.5478	401.0147	1.9904	0.3112	0.1763	0.0509
EE	267.4865	386.4471	386.6614	390.6021	388.0690	0.8708	0.1442	0.1148	0.4180
E	304.9673	611.9345	612.0060	613.9950	612.7371	1.7555	0.3236	0.3869	0.0000
MOE	274.3689	552.7378	552.9560	556.8587	554.3430	2.2232	0.4015	0.1498	0.1481
EW	266.2673	538.5346	538.9790	544.7159	540.9423	0.9065	0.1651	0.1148	0.4282
OWE	199.4381	404.8762	405.3125	411.1088	407.3091	2.1691	0.3368	0.1446	0.1694
W	197.2967	398.5934	398.8077	402.7485	400.2154	1.8483	0.2897	0.1392	0.2025
TLE	191.2235	386.4471	386.6614	390.6021	388.0690	0.8708	0.1442	0.1148	0.4183

**Table 5:** Estimates of $VaR_{q}$ for D1 for selected values of *q*.

*q*	TBXE	BX	EE	EW	W	E
0.55	78.32	42.40	68.33	67.06	61.48	56.43
0.60	84.53	46.69	71.52	70.33	68.02	64.76
0.65	91.98	51.44	74.99	73.95	75.17	74.20
0.70	100.92	56.81	78.84	78.04	83.13	85.09
0.75	112.17	63.03	83.23	82.80	92.21	97.98
0.80	127.27	70.52	88.43	88.57	102.89	113.75
0.85	149.78	80.02	94.94	95.99	116.11	134.08
0.90	191.17	93.20	103.85	106.51	133.86	162.74
0.95	357.62	115.42	118.67	124.94	162.42	211.72

**Table 6:** Estimates of $ES_{q}$ for D1 for selected values of *q*.

*q*	TBXE	BX	EE	EW	W	E
0.55	51.68	18.44	51.84	51.27	31.64	24.50
0.60	54.16	19.86	53.34	52.72	34.40	27.50
0.65	56.79	21.33	54.87	54.21	37.26	30.72
0.70	59.63	22.88	56.45	55.77	40.24	34.21
0.75	62.76	24.53	58.08	57.41	43.40	38.02
0.80	66.31	26.31	59.81	59.17	46.77	42.24
0.85	70.53	28.27	61.68	61.10	50.45	47.01
0.90	75.96	30.51	63.76	63.31	54.56	52.59
0.95	84.91	33.21	66.21	66.01	59.41	59.53

**Table 7:** Estimates of $VaR_{q}$ for D2 for selected values of *q*.

*q*	TBXE	BX	EE	EW	W	E
0.55	69.34	6.37	12.76	0.10	13.58	10.78
0.60	75.44	7.30	13.60	0.12	14.64	12.37
0.65	82.31	8.34	14.51	0.13	15.78	14.17
0.70	90.22	9.55	15.53	0.15	17.01	16.25
0.75	99.57	10.97	16.70	0.18	18.38	18.71
0.80	110.98	12.71	18.09	0.20	19.95	21.72
0.85	125.59	14.95	19.83	0.24	21.83	25.60
0.90	145.60	18.10	22.23	0.28	24.28	31.07
0.95	176.39	23.49	26.23	0.35	28.06	40.43

**Table 8:** Estimates of $ES_{q}$ for D2 for selected values of *q*.

*q*	TBXE	BX	EE	EW	W	E
0.55	42.77	2.80	8.59	0.04	8.01	4.68
0.60	45.23	3.14	8.98	0.05	8.51	5.25
0.65	47.82	3.50	9.37	0.05	9.03	5.87
0.70	50.56	3.88	9.77	0.06	9.55	6.53
0.75	53.50	4.31	10.19	0.07	10.10	7.26
0.80	56.73	4.78	10.64	0.07	10.66	8.07
0.85	60.33	5.31	11.13	0.08	11.26	8.98
0.90	64.48	5.92	11.67	0.09	11.91	10.04
0.95	69.49	6.69	12.32	0.10	12.65	11.37

## Data Availability

The data that support the findings of this study are available from the corresponding author, upon reasonable request.
